# Cruzipain Promotes *Trypanosoma cruzi* Adhesion to *Rhodnius prolixus* Midgut

**DOI:** 10.1371/journal.pntd.0001958

**Published:** 2012-12-13

**Authors:** Lívia Almeida Uehara, Otacílio C. Moreira, Ana Carolina Oliveira, Patrícia Azambuja, Ana Paula Cabral Araujo Lima, Constança Britto, André Luis Souza dos Santos, Marta Helena Branquinha, Claudia Masini d'Avila-Levy

**Affiliations:** 1 Laboratório de Biologia Molecular e Doenças Endêmicas, Instituto Oswaldo Cruz (IOC), Fundação Oswaldo Cruz (FIOCRUZ), Rio de Janeiro, Brazil; 2 Instituto Federal de Educação, Ciência e Tecnologia do Rio de Janeiro, Rio de Janeiro, Brazil; 3 Laboratório de Imunologia Molecular, Instituto de Biofísica Carlos Chagas Filho (IBCCF), Universidade Federal do Rio de Janeiro, Rio de Janeiro, Brazil; 4 Laboratório de Bioquímica e Fisiologia de Insetos, Instituto Oswaldo Cruz (Fiocruz), Rio de Janeiro, Brazil; 5 Laboratório de Bioquímica e Biologia Molecular de Proteases, Instituto de Biofísica Carlos Chagas Filho (IBCCF), Universidade Federal do Rio de Janeiro, Rio de Janeiro, Brazil; 6 Laboratório de Estudos Integrados em Bioquímica Microbiana, Departamento de Microbiologia Geral, Instituto de Microbiologia Paulo de Góes (IMPG), Universidade Federal do Rio de Janeiro, Rio de Janeiro, Rio de Janeiro, Brazil; 7 Laboratório de Bioquímica de Proteases, Departamento de Microbiologia Geral, Instituto de Microbiologia Paulo de Góes (IMPG), Universidade Federal do Rio de Janeiro, Rio de Janeiro, Rio de Janeiro, Brazil; National Institutes of Health, United States of America

## Abstract

**Background:**

*Trypanosoma cruzi* is the etiological agent of Chagas' disease. Cysteine peptidases are relevant to several aspects of the *T. cruzi* life cycle and are implicated in parasite-mammalian host relationships. However, little is known about the factors that contribute to the parasite-insect host interaction.

**Methodology/Principal Findings:**

Here, we have investigated whether cruzipain could be involved in the interaction of *T. cruzi* with the invertebrate host. We analyzed the effect of treatment of *T. cruzi* epimastigotes with anti-cruzipain antibodies or with a panel of cysteine peptidase inhibitors (cystatin, antipain, E-64, leupeptin, iodocetamide or CA-074-OMe) on parasite adhesion to *Rhodnius prolixus* posterior midgut *ex vivo*. All treatments, with the exception of CA074-OMe, significantly decreased parasite adhesion to *R. prolixus* midgut. Cystatin presented a dose-dependent reduction on the adhesion. Comparison of the adhesion rate among several *T. cruzi* isolates revealed that the G isolate, which naturally possesses low levels of active cruzipain, adhered to a lesser extent in comparison to Dm28c, Y and CL Brener isolates. Transgenic epimastigotes overexpressing an endogenous cruzipain inhibitor (pCHAG), chagasin, and that have reduced levels of active cruzipain adhered to the insect gut 73% less than the wild-type parasites. The adhesion of pCHAG parasites was partially restored by the addition of exogenous cruzipain. *In vivo* colonization experiments revealed low levels of pCHAG parasites in comparison to wild-type. Parasites isolated after passage in the insect presented a drastic enhancement in the expression of surface cruzipain.

**Conclusions/Significance:**

These data highlight, for the first time, that cruzipain contributes to the interaction of *T. cruzi* with the insect host.

## Author Summary

Chagas' disease, a neglected tropical disease caused by *Trypanosoma cruzi*, is transmitted to vertebrate hosts by hematophagous insects. Cruzipain is a lysosomal cysteine peptidase, which plays an important role in parasite infectivity, intracellular growth and differentiation, and is abundantly expressed on the surface of epimastigotes. Since these forms face the insect vector environment during the life cycle, it is conceivable that cruzipain may participate in the interaction process with the invertebrate host. Here, we showed that adhesion of *T. cruzi* to the insect midgut cells was inhibited by the blockage of cruzipain function. Cysteine peptidase inhibitors, in a dose-dependent manner, and anti-cruzipain antibodies were able to reduce the binding of epimastigote forms to the *Rhodnius prolixus* midgut. Similarly, *T. cruzi* transfectants that overexpress chagasin, the endogenous cruzipain inhibitor, displayed low levels of adhesion. Accordingly, the supplementation of exogenous cruzipain partially restored the adherence of the transfected line. Additionally, the ability of the chagasin overexpressing transfectants to colonize the insect *in vivo* was drastically reduced, and the levels of cruzipain expression by wild-type parasites were enhanced after *in vivo* passage in *R. prolixus.* Collectively, our results strongly suggest that cruzipain is required for successful colonization of *R. prolixus* by *T. cruzi*.

## Introduction

Chagas' disease remains one of the most important neglected diseases of Latin America, and it has become a world health problem due to migration of infected people from endemic countries [1]. Every year, it is estimated that 14,000 people die as consequence of the infection in Latin America [2]. The etiological agent *Trypanosoma cruzi* is transmitted in nature to vertebrate hosts through hematophagous insects from the Reduviidae family. During their development within insects, the parasites undergo profound morphological changes, modulating surface molecules to enable interactions with specific insect tissues that are essential for their survival, development and successful transmission to the vertebrate host. *T. cruzi-*insect vector interaction begins when the insect feeds on blood of an infected vertebrate host. Once ingested, most of the bloodstream trypomastigotes differentiate into a mammalian non-infective epimastigote forms. In the posterior midgut, they divide repeatedly by binary fission and adhere to perimicrovillar membranes (PMM) of the intestinal cells. In the rectum, where the highest parasite population occurs, a proportion of epimastigotes attach to the rectal cuticle by hydrophobic interactions and transforms into non-replicative metacyclic trypomastigotes (metacyclogenesis), which are eliminated with the feces and urine during blood feeding, infecting another mammalian host [3]. In this scenario, the PMMs act as an adhesion site, becoming essential to the establishment of the parasite in the insect vector. In addition, *T. cruzi-*PMM interaction appears to be necessary for metacyclogenesis, but there is a general lack of information regarding which parasite molecules are implicated in this process [3], [4].

Cruzipain, a member of the papain superfamily, is a cysteine peptidase of *T. cruzi* that is an important virulence factor of this parasite, which is involved in several crucial steps in the interaction with mammalian cells, such as in the host cell invasion, and parasite survival, differentiation and multiplication within host cell [4]–[12]. The involvement of cruzipain in the metacyclogenesis process has been indirectly demonstrated by several approaches [4], [11], [12]. The participation of cruzipain in host cell invasion by trypomastigotes is mediated through at least two distinct pathways [8], [9]. One pathway involves the triggering of the B2 type of bradykinin receptor (B2R), whereas the other pathway is independent of the kinin receptors [8], [9]. More recently, it was uncovered that cruzipain also participates in the mobilization of endothelin receptors during the invasion of smooth muscle [13]. Also, cruzipain can cleave at the hinge of all human IgG subclasses, which might be relevant to parasite escape from the adaptive immune response [14]. The drug candidate, N-methyl-piperazine-Phe-homoPhe-vinyl sulphone phenyl (K777), a potent cruzipain inhibitor, is in late preclinical trials for Chagas' disease chemotherapy. This drug rescued mice from a lethal infection of *T. cruzi*, promoting parasitological cure in most of them, even in an immunodeficient mouse model [15].

Cruzipain is expressed at variable levels in all developmental forms and strains of the parasite, being abundantly detected in epimastigote forms. This enzyme is found in the endosomal-lysosomal system of epimastigote (especially in reservosomes), amastigote and trypomastigote forms, and is profusely detected on the surface of epimastigotes and amastigotes [5]. Some isoforms are associated to the plasma membrane of epimastigotes, presumably through a glycosylphosphatidylinositol (GPI) anchor [16]. There is a common notion that cruzipain, as the major lysosomal peptidase of *T. cruzi*, may play a prominent role in nutrition of the parasite, at least in the gut of the hematophagous insect vector, however, up to now, no experimental evidence supports such concept. In *T. cruzi*, a potent endogenous cruzipain inhibitor, chagasin, forms tight binding complexes with the enzyme *in vivo*, regulating its activity. Parasites lines transfected with an episomal plasmid containing the chagasin gene express four-fold more chagasin than wild-type parasites and exhibit 70–80% reduction in the overall cysteine peptidase activity [10]. These transfectants provide an interesting tool to asses cruzipain function. For instance, it was shown that those lines have reduced capacity to differentiate into trypomastigotes, as well as reduced infectivity to mammalian cells *in vitro* [10].

In the present study, we sought to investigate whether *T. cruzi* cruzipain might be involved in the interaction of epimastigotes with *R. prolixus* midgut. For this purpose, we analyzed the effects of anti-cruzipain antibodies, as well as, of a panel of cysteine peptidase inhibitors on the parasite adhesion to *R. prolixus* posterior midgut *ex vivo*. We also compared the adhesion rate to the insects among chagasin transfectants and wild-type parasites, both *ex vivo* or *in vivo*. Our findings point to a prominent role for cruzipain in the interaction of *T. cruzi* with the invertebrate host.

## Methods

### Parasite culture

*Trypanosoma cruzi*, Dm28c (COLPROT 010), G (COLPROT 216), Y (COLPROT 106) and CL Brener (COLPROT 005) isolates, obtained from the *Coleção de Protozoários da Fundação Oswaldo Cruz (COLPROT-FIOCRUZ)*, were used in this work. The transgenic lines were obtained using the episomal pTEX shuttle vector containing the chagasin-encoding gene (pCHAG). A parasite line harboring the empty vector (pTEX) was also used in parallel for control [10]. The epimastigote forms of *T. cruzi* were grown in 3.7% brain heart infusion medium (BHI), containing 0.002% hemin, supplemented with 10% heat-inactivated fetal bovine serum (FBS), at 28°C for 4 days, to reach late-log growth phase. The transgenic parasites were maintained in BHI, supplemented with 800 µg/mL geneticin. For all experiments, epimastigotes were harvested by centrifugation (1500× *g* for 5 min at 25°C), washed three times in 0.15 M NaCl, 0.01 M phosphate-buffer pH 7.2 (PBS) and immediately used.

### Insects

*Rhodnius prolixus* were reared and maintained as previously described [17]. Briefly, fifth-instars larvae were randomly chosen, starved for 30 days after the last ecdysis and then allowed to feed on defibrinated rabbit blood through a membrane feeder. Ten days after feeding, the insects were dissected; the posterior midguts removed, longitudinally sectioned and washed three times in PBS to expose their luminal surfaces [18]. After the washing, the tissue fragments were processed as described below. The insects were obtained from the insectary of the Laboratório de Bioquímica e Fisiologia de Insetos, Instituto Oswaldo Cruz, FIOCRUZ.

### Ex vivo interaction between *R. prolixus* dissected midgut and *T. cruzi*

For the interaction assays, the tissue fragments were placed into Eppendorf microtubes and then, incubated with the parasites (2.0×10^7^ in 100 µL of PBS) for 15 min at room temperature, under gentle shaking. Only one dissected midgut was added to parasites per treatment. Wild-type Dm28c parasites were pre-treated or not for 1 h with a panel of different cysteine peptidase inhibitors: iodoacetamide, leupeptin, antipain, CA-074-OMe [L-3-*trans*-(propylcarbamoyl)oxirane-2-carbonyl]-L-isoleucyl-L-proline methyl ester or E-64 [(*trans*-(epoxy-succinyl)-L-leucylamino-(4-guanidino)butane] at 10 µM, or chicken egg white cystatin at 1 µg/mL. For the dose-dependent assay, we used cystatin concentrations ranging from 0.1 to 10 µg/µL. Wild-type parasites were treated with anti-cruzipain antibodies at 1∶1000 or 1∶2500 dilution or with rabbit pre-immune serum (1∶1000) for 1 h. The viability of the parasites throughout the experiment was assessed by mobility and trypan blue dye exclusion. After each treatment, parasites were washed three times with PBS prior to the interaction assays. Alternatively, the adhesion rate of several *T. cruzi* isolates was compared: wild-type parasites, pTEX and pCHAG (Dm28c), as well as G, Y and CL Brener. After incubation with the parasites, the explanted midguts were spread onto glass slides and the numbers of attached parasites per 100 randomly chosen epithelial cells in 10 different fields of each midgut explanted were quantified by counting under the light microscope [18]. Results are shown as the mean ± standard error of two experiments performed in quadruplicate.

### *Ex vivo* interaction between *R. prolixus* midgut and epimastigotes in the presence of exogenous cruzipain

In this set of experiments, tissue fragments were pre-treated for 15 min at room temperature with 1.5 µg/µL of exogenous active cruzipain obtained from *T. cruzi* Dm28c epimastigotes as previously described [19] or heat-inactivated cruzipain. After this treatment, the midguts were gently washed in PBS and the interaction with Dm28c epimastigotes was performed as described before. Alternatively, pCHAG parasites were incubated for 15 min at room temperature with *R. prolixus* dissected midguts in the absence or presence of increasing concentrations of exogenous active cruzipain ranging from 1.875 to 7.5 µg/µL. The interaction process was carried out as described above.

### *In vivo* colonization of *R. prolixus* by *T. cruzi*

After a starvation period of 30 days, fifth-instars larvae were fed through a membrane feeder on defibrinated rabbit blood containing 9×10^6^ *T. cruzi* cells/mL (wild-type, pTEX or pCHAG). Twenty days after infection, the entire posterior midgut of 4 insects or the entire rectum of 8 insects were obtained, pooled and gently homogenized in 1 mL of PBS. Then, two aliquots from the homogenate were used to quantify in a hemocytometer chamber the total number of live flagellates. The same tissue preparations were also used for parasite quantification through real-time PCR assays, as described below.

### Assessment of *in vivo* colonization through real time PCR

A pool of 4 midguts or a pool of 8 recta were resuspended in 100 µL of 10 mM Tris-HCl, 1 mM ethylenediaminetetraacetic acid (EDTA) buffer, pH 8.0 (TE buffer) containing 100 µg/mL proteinase K and incubated for 2 h at 56°C. Then, total DNA was extracted using Wizard Genomic DNA Purification Kit (Promega) according to the manufacturer's instructions, with slight modifications. Briefly, after the treatment with lysis buffer, the samples were centrifuged at 2,000× *g* for 5 min, the collected supernatant was incubated at 56°C for 2 min to proceed the following steps. At the final step, the DNA was eluted with 200 µL of ultrapure water, and incubated at 25°C for 10 min. The purity (A260/280 nm ratio) and the concentration of DNA were estimated by spectrophotometry using a NanoDrop (Thermo Scientific). After that, absolute quantification of *T. cruzi* in each sample was performed through real-time quantitative PCR in a thermocycler ABI Prism 7500 Fast Sequence Detection System (Applied Biosystems, Foster City, CA, USA). The quantification was performed in a final volume of 20 µL containing: 2 µL DNA, 10 µL 2× Power SYBR Green master mix (Applied Biosystems, CA), 0.3 µM primers for the *T. cruzi* satellite DNA region [20] or 0.1 µM primers for the *R. prolixus* 12S ribosomal RNA gene. Primers used for *T. cruzi* and insect DNA sequences were, respectively: Cruzi 1 (Forward) 5′-ASTCGGCTGATCGTTTTCGA-3′, Cruzi 2 (Reverse) 5′-AATTCCTCCAAGCAGCGGATA-3′, P2b (Forward) 5′-AAAGAATTTGGCGGTAATTTAGTCT-3′ and P6 (Reverse) 5′-GCTGCACCTTGACCTGACATT-3′. The PCR conditions were: 50°C for 2 min, 95°C for 10 min followed by 40 cycles at 95°C for 30 sec and 58°C for 1 min. Parasites were quantified using the absolute quantification method, and samples were normalized to the *R. prolixus* 12S ribosomal RNA gene. The standard curves were prepared from parasite DNA serially 10-fold diluted in TE buffer.

### Determination of surface cruzipain after *in vivo* colonization of *R. prolixus*

For parasite re-isolation, a pool of 8 recta from infected insects was resuspended in 1 mL of PBS and centrifuged at 300× *g* for 1 min. The supernatant was discharged and the pellet was incubated with 500 µL of PBS for 30 min at 28°C. The supernatant was collected and centrifuged again at 1500× *g* for 5 min. The pellet contained *T. cruzi* cells and, in minor quantity, small fragments of the insect rectum. Finally, re-isolated cells from the rectum (at least 10^6^ cells) were fixed in 0.1% paraformaldehyde in PBS (pH 7.2) for 30 min at 26°C, followed by extensive washing in the same buffer. The fixed cells maintained their morphological integrity, as verified by optical microscopic observation. After this step, the cells were incubated for 1 h at room temperature with a 1∶1000 dilution of the anti-cruzipain antibody. Cells were then incubated for an additional hour with a 1∶100 dilution of fluorescein isothiocyanate (FITC)-labeled goat anti-rabbit IgG. The cells were then washed 3 times in PBS and the parasite associated fluorescence was quantified in a flow cytometer (FACSCalibur, BD Bioscience, USA) equipped with a 15 mW argon laser emitting at 488 nm. Non-treated parasite cells from culture or from insect rectum and cells treated with the secondary antibody alone were run in parallel for control. Each experimental population was then mapped by using a two-parameter histogram of forward-angle light scatter versus side scatter. The mapped population (n = 10,000) was then analyzed for log green fluorescence by using a single parameter histogram [21].

### Statistical analysis

All *ex vivo* experiments were repeated two times in quadruplicate. All *in vivo* infection assays, including qPCR assays, were performed as three independent experiments in triplicate. The data was analyzed statistically by means of Student's *t* test, or the analysis of variance between groups was performed by means of ANOVA test using EPI–INFO 6.04 (Database and Statistics Program for Public Health) computer software. *P* values of 0.05 or less were considered statistically significant.

## Results

### Effect of cysteine peptidase inhibitors and anti-cruzipain antibodies on the *T. cruzi-R. prolixus* interaction

**Figure 1:**
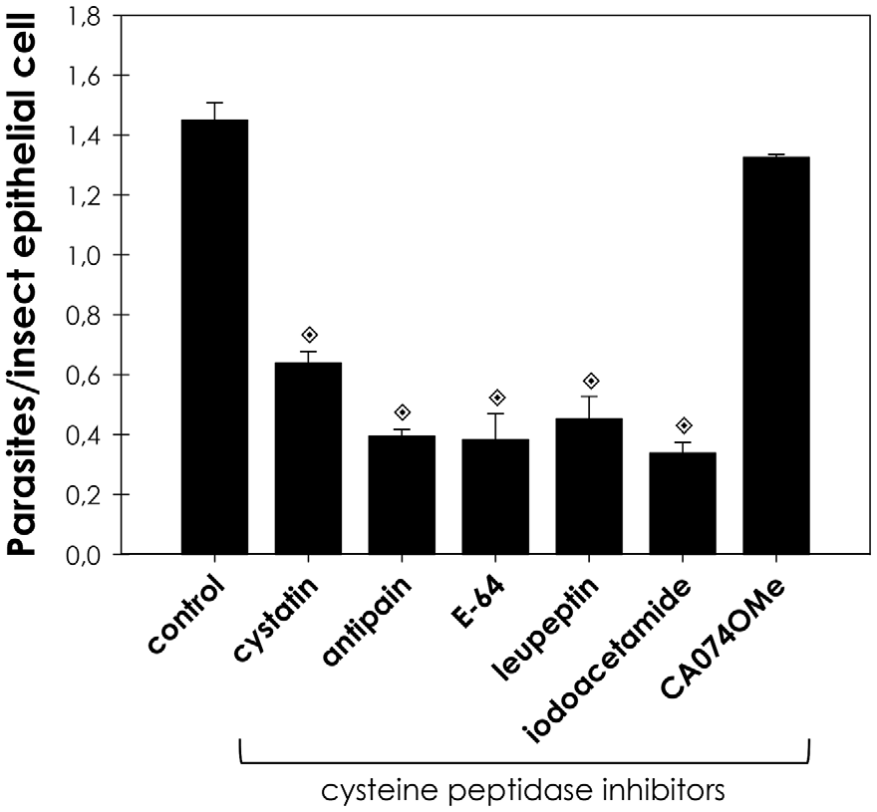
Cysteine peptidase inhibitors reduce the interaction between *T. cruzi* and *R. prolixus* midgut. The parasites (2.0×10^7^ cells) were treated for 60 min at room temperature with chicken egg white cystatin (1 µg/mL), antipain, E-64, leupeptin, iodocetamide or CA-074-OMe, at 10 µM. The viability of the parasites was not affected by the treatments used in this set of experiments. Following interaction for 15 min with the insect gut, the number of adhered parasites/insect gut epithelial cells was estimated by randomly counting at least 100 epithelial cells in quadruplicate. The results are shown as the mean ± SEM of two independent experiments. The symbol indicates systems significantly different from untreated (control) cells by means of Students' *t* test (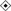, *P*<0.001).

In order to evaluate whether cysteine peptidase inhibitors influence the adhesion of *T. cruzi* to dissected *R. prolixus* posterior midgut, we performed experiments in which each cysteine peptidase inhibitors were incubated with epimastigote forms, followed by their exposure to dissected *R. prolixus* posterior midgut. The parasites maintained their viability under this condition, as judged by their mobility and trypan blue dye exclusion. After this time, untreated *T. cruzi* epimastigotes were allowed to bind to dissected *R. prolixus* posterior midgut, revealing many parasites adhered to the insect epithelial cells mainly by their flagella. Our results showed that iodoacetamide, leupeptin, antipain, E-64 and cystatin significantly reduced, on average 70%, the adhesion of *T. cruzi* to *R. prolixus* posterior midgut in relation to untreated parasites (Fig. 1). Considering the presence of a significant activity of a 30-kDa cathepsin B like-cysteine peptidase in *T. cruzi* extracts [22], we analyzed the effect of CA-074-OMe, a specific inhibitor of cathepsin B. Our results revealed that this inhibitor showed no significant change in the interaction process (Fig. 1).

**Figure 2:**
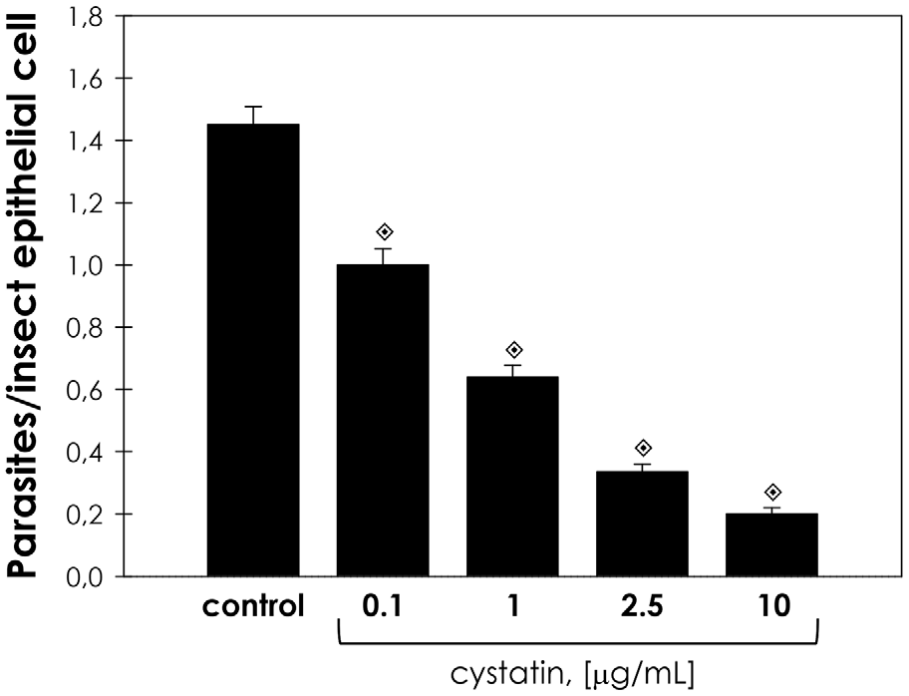
Dose dependent effect of cystatin on the interaction between *T. cruzi* and *R. prolixus* midgut. Epimastigotes (2.0×10^7^ cells) were treated for 60 min at room temperature with different concentrations of cystatin from chicken egg white (0.1, 1, 2.5 or 10 µg/mL). The viability of the parasites was not affected by the treatments used in this set of experiments. Following interaction for 15 min with the insect gut, the number of parasites/insect gut epithelial cells was estimated by randomly counting at least 100 epithelial cells in quadruplicate. The results are shown as the mean ± SEM of two independent experiments. Symbols indicate systems significantly different from untreated (control) cells by means of Students' *t* test (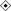, *P*<0.001).

**Figure 3:**
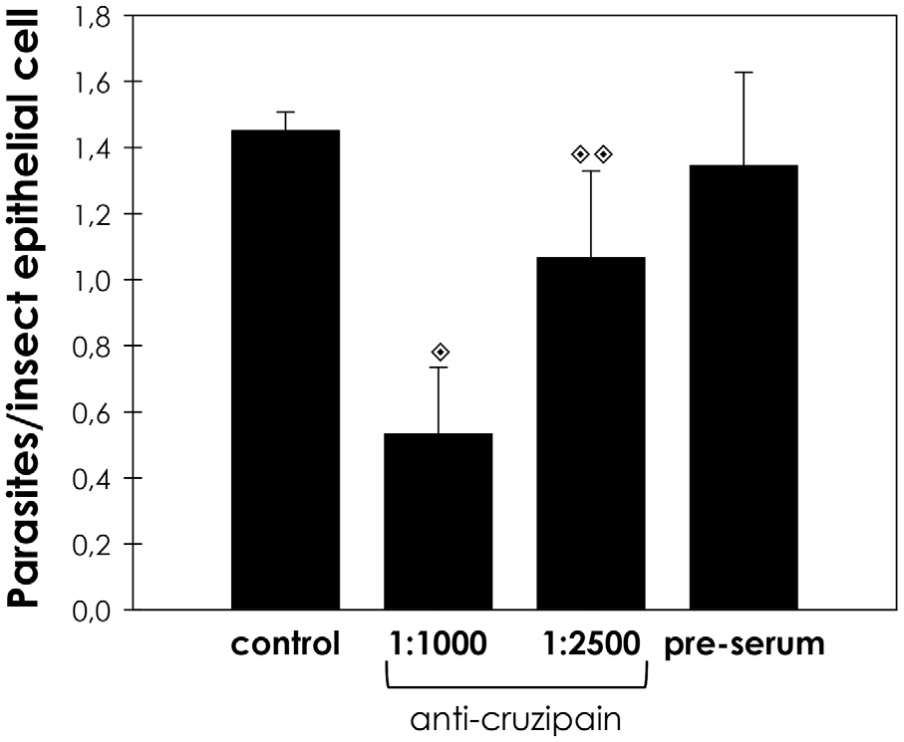
Antibodies to cruzipain affect the interaction between *T. cruzi* and *R. prolixus* midgut. The epimastigotes (2.0×10^7^ cells) were treated for 60 min at room temperature with anti-cruzipain antibodies at 1∶1000 or 1∶2500 dilution, or pre-immune serum at 1∶1000. The viability of the parasites was not affected by the treatment used in this set of experiments. Following interaction for 15 min with the insect gut, the number of adhered parasites/insect gut epithelial cells was estimated by randomly counting at least 100 epithelial cells in quadruplicate. The results are shown as the mean ± SEM of two independent experiments. Symbols indicate systems significantly different from untreated (control) cells by means of Students' *t* test (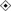, *P = *0.000802; 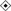 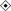, *P* = 0.004351).

Cystatin is a high affinity tight binding inhibitor of cathepsin L cysteine peptidases, such as cruzipain. Therefore, we evaluated the effect of increasing concentrations of cystatin on the proportion of parasite adhesion to midgut. In cystatin doses ranging from 0.1 to 10 µg/mL, the adhesion of epimastigotes diminished from 73% to 15% in relation to the control (Fig. 2). Supporting the hypothesis that parasite cysteine peptidases play a role in the adhesion to the insect, the pre-treatment of parasites with anti-cruzipain antibodies considerably reduced the interaction process, in relation to the control (Fig. 3). The antibody concentrations used did not promote parasite agglutination (data not shown). Parasites treated with the pre-immune serum adhered to the midguts at a rate similar to that of the control (Fig. 3).

### Evaluation of cruzipain involvement in the interaction with *R. prolixus* using chagasin-transfectants

**Figure 4:**
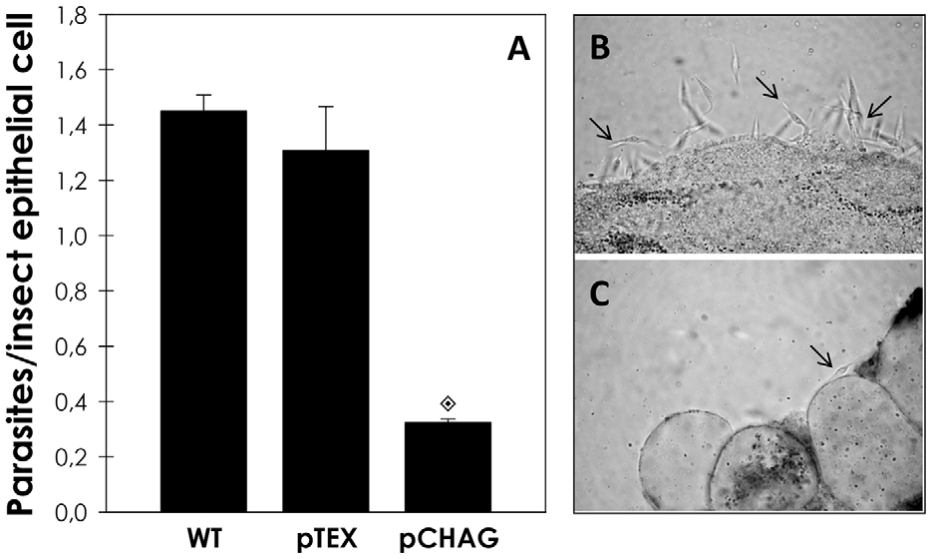
*T. cruzi* chagasin overexpressing transfectants present reduced interaction rate with *R*. *prolixus* midguts. Epimastigotes (2.0×10^7^ cells) wild-type, or transfected with empty vector (pTEX) or with the vector containing the chagasin gene (pCHAG) were incubated with the dissected guts of the triatomine for 15 min at room temperature. The number of adhered parasites/insect gut epithelial cells was estimated by randomly counting at least 100 epithelial cells in quadruplicate. Results are shown as the mean ± SEM of two independent experiments. Parasites pCHAG showed adhesion rate significantly different from the wild-type and of control pTEX parasites by means of Students' *t* test (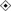, *P<*0.001) (A). The insets show phase-contrast photomicrography of *T.* cruzi wild-type (B) or pCHAG (C) incubated *ex vivo* with dissected posterior midgut epithelial cells of *R. prolixus*, 10 days after the blood meal. 4.500×· The arrows in (B) and (C) indicate the parasites attached to the epithelial cells.

**Figure 5:**
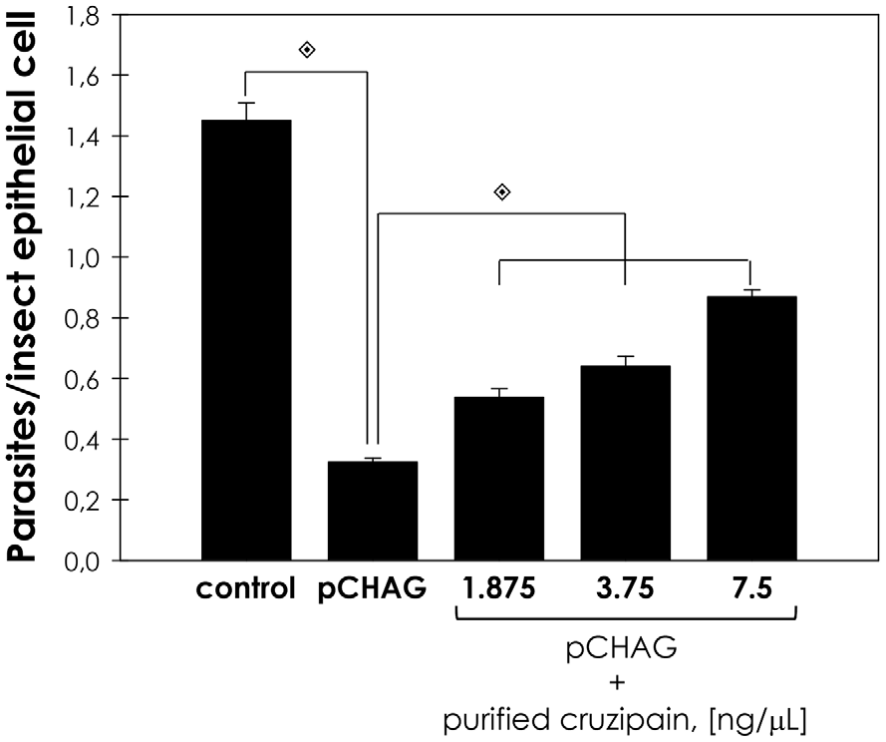
Exogenous cruzipain restores the interaction of chagasin overexpressing transfectants with *R. prolixus* midguts. Exogenous cruzipain was supplemented in the interaction media at 1.875, 3.75 and 7.5 ng/µL. Following interaction for 15 min with the insect gut, the number of parasites/insect gut epithelial cells was estimated by randomly counting at least 100 epithelial cells in quadruplicate. The results are shown as the mean ± SEM of two independent experiments. Parasites pCHAG supplemented with exogenous cruzipain had an adhesion rate significantly different from the parasites pCHAG using Students' *t* test (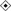, *P<*0.001).

Transgenic parasite lines overexpressing chagasin present a four-fold increase in cysteine peptidase inhibitory activity and reduced levels of active cruzipain, posing as a tool to address the role of this parasite peptidase in the interaction with the insect vector [10]. Chagasin overexpressing line (pCHAG) displayed low rates (73% lower than control) of adhesion to insect dissected midguts *ex vivo*. Parasites carrying empty vector (pTEX) were used as controls and did not show any significant alteration in the adhesion rate in relation to wild-type parasites (Fig. 4). In order to assess if the diminished capacity of pCHAG to adhere was related to reduced cruzipain activity, we added exogenous cruzipain to the interaction media. In this condition, the adhesion of pCHAG raised systematically from 27% (no supplementation) up to 60% in relation to the control, as function of exogenous cruzipain concentration supplemented to the assay (Fig. 5).

### Evaluation of the adhesion of different *T. cruzi* isolates

**Figure 6:**
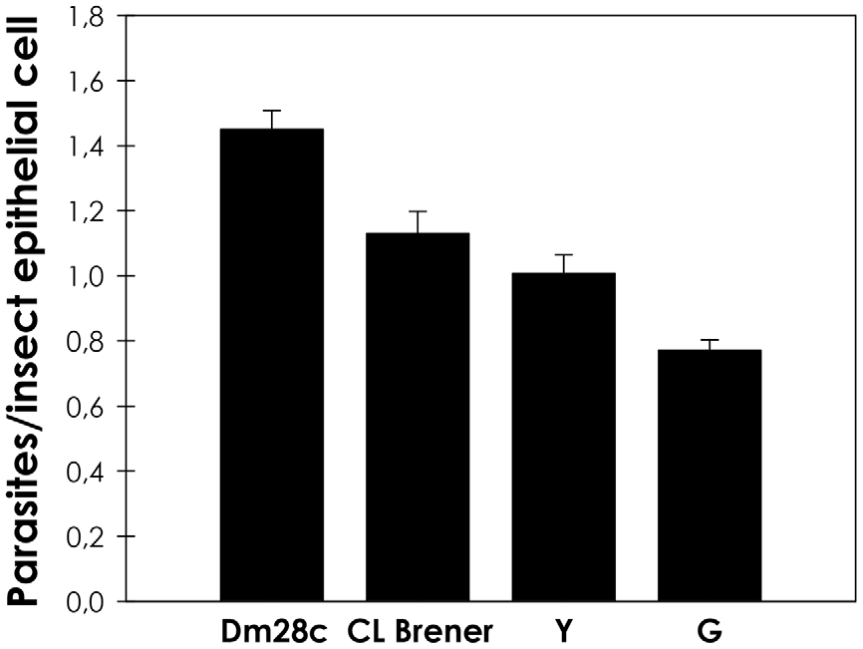
Interaction rate of different *T. cruzi* isolates with *R*. *prolixus* midguts. Epimastigote forms (2.0×10^7^ cells) of Dm28c, CL-Brener, Y and G strains were incubated with the dissected guts of the triatomine for 15 minutes at room temperature. Following, the number of parasites/insect gut epithelial cells was estimated by randomly counting at least 100 epithelial cells in quadruplicate. The results are shown as the mean ± SEM of two independent experiments. G strain had an adhesion rate significantly different from the others *T. cruzi* isolates by means of ANOVA variance (*P*≤0.05).

It is known that distinct *T. cruzi* isolates have natural differences in the stoichiometric balance of cruzipain:chagasin [10], which impact on the overall cysteine peptidase activity of the parasite. In this sense, the adhesion rate to *R. prolixus* dissected midguts was compared, revealing a correlation with the overall cysteine peptidase activity. Our results demonstrated that the G isolate, which presents ten times less cruzipain activity than Dm28c [10], presented the lower capability to adhere to the insect luminal midgut surface of *R. prolixus* in relation to the other isolates (Fig. 6).

### Effect of pre-treatment of *R. prolixus* dissected midgut with active cruzipain

In order to assess if cruzipain could act as a direct ligand for possible receptors in the insect epithelial midgut cells, as previously described for the gp63 (a metallopeptidase) from a lower trypanosomatid in the adhesion to an insect host model [23], we tested the effect of the pre-treatment of dissected midguts with cruzipain molecules. Our results showed that the pre-incubation of either active or heat-inactivated cruzipain did not promote any significant alteration in the interaction rate (data not shown).

### *R. prolixus in vivo* infection assay with chagasin overexpressing *T. cruzi*

**Figure 7:**
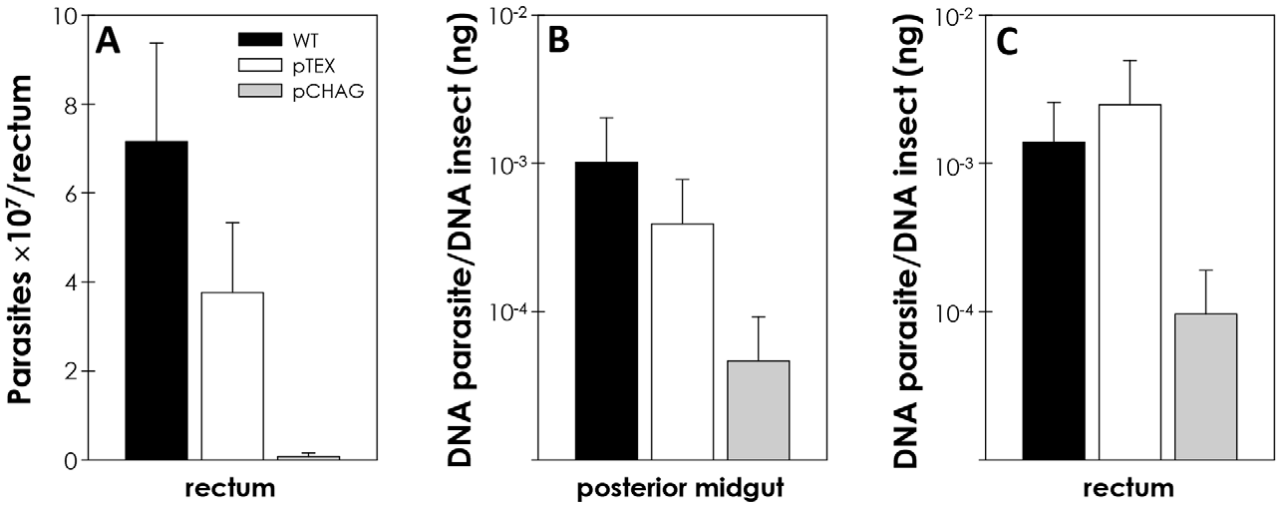
*In vivo* infection of *R*. *prolixus* by *T. cruzi* is reduced upon chagasin overexpression. The insects were fed with defibrinated rabbit blood containing 9×10^6^ parasites/mL (Wild-type, pTEX, pCHAG) *ad libitum.* Twenty days later, intact pools of 4 midguts or 8 recta were excised from the insects, and processed as described in Methods section. The parasites were quantified by the following methods: (A) microscopic counts of the infection levels of *T. cruzi* in the rectum of *R*. *prolixus*, (B, C) quantitative PCR (qPCR) for estimating the infection levels of *T. cruzi* in the midgut or rectum of *R*. *prolixus*. The results are shown as the mean ± SEM of three independent experiments. Parasites pCHAG showed a rate of adhesion to the cells not statistically different from control and pTEX by means of Students' *t* test, due to intrinsic biological triatomine variance.

In order to compare the infection levels *in vivo* of epimastigote forms of *T. cruzi* (wild-type, pTEX and pCHAG), insects were fed with defibrinated rabbit blood containing parasites. Twenty days after blood feeding, the insect midguts and recta were screened for parasites by direct microscopic counting. Parasites were only detected in the rectum. As expected, chagasin-transfectants (pCHAG) displayed low rates of colonization in comparison to both wild-type and pTEX parasites (Fig. 7A). Although the possible participation of cruzipain on the colonization process was demonstrated through this approach, the absence of parasites in the midgut was unexpected, and led us to develop, for the first time, a methodology using quantitative real-time PCR (qPCR), targeting *T. cruzi* satellite-DNA, to quantify with higher sensibility and accuracy *T. cruzi* infection in *R. prolixus* midgut and rectum. The samples were normalized to the *R. prolixus* 12S ribosomal RNA gene. qPCR assays revealed that control parasites were detected both in the midgut and rectum, being more abundant in the latter, while pCHAG parasites were detected at considerably lower levels (Fig. 7B,C).

### Modulation of cruzipain levels after *R. prolixus* colonization

**Figure 8:**
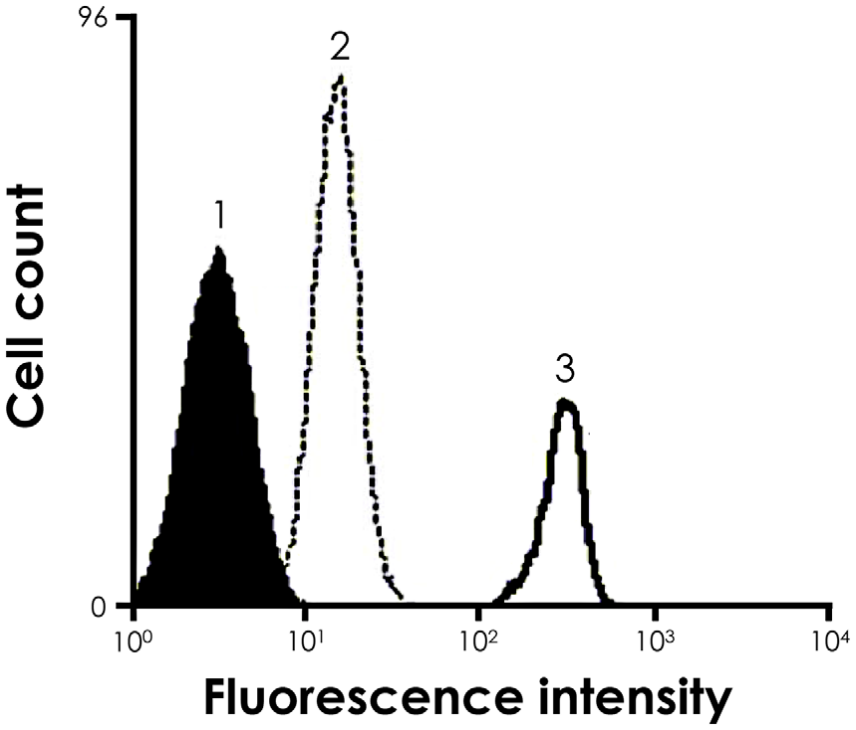
Cruzipain expression in *T. cruzi* cells is enhanced after passage in *R. prolixus*. Cells re-isolated after the colonization (3) or cells obtained from cultivation in axenic medium (BHI) (2) were incubated in the presence or absence (1) of anti-cruzipain antibodies at 1∶2000 dilution and then analyzed by flow cytometry. Representative data of the analysis of 10,000 cells from one of three experiments are shown. The curve 3 represents 10,000 events positive for cruzipain.

In order to assess the levels of cruzipain expression of *T. cruzi* after passage in *R. prolixus*, parasites were re-isolated after the colonization and the levels of anti-cruzipain binding to the parasite surface was compared through flow cytometry with cells obtained from cultivation in axenic medium (BHI). Our data revealed that, after colonization of the insect host rectum, parasites demonstrated a significant increase in the surface cruzipain expression (Fig. 8).

## Discussion

Pathogenic protozoa express large amounts and varied patterns of intracellular and/or extracellular peptidases that are involved in specific and extremely necessary functions in the parasite life-cycle, either directly through its catalytic properties or indirectly by regulating other proteins [24]. Cysteine peptidases from the papain superfamily of kinetoplastid parasites are considered as key factors for survival and interaction with the vertebrate host. Due to the importance of peptidases in physiological processes, they have emerged as promising targets for antiparasitic drugs. Therefore, *T. cruzi* cruzipain has been extensively investigated as a target for Chagas' disease chemotherapy [15], [25]. Although *T. cruzi* cruzipain is expressed abundantly on the surface of epimastigote forms, found in the insect vector, its role in parasite interaction with the insect has never been addressed before. Our research group has been studying some peptidases believed to be essential in this part of the life cycle of trypanosomatids, like gp63 and cruzipain [21], [23], [26]–[28]. Cruzipain homologues have been described in insect and plant trypanosomatids, namely *Blastocrithidia culicis* (recently reassigned as *Strigomonas culicis* [29]) and *Phytomonas serpens*, respectively [21], [27], [28]. In the latter, it was shown that cruzipain-like proteins are located at *P. serpens* cell surface, and are implicated on the adhesion to the salivary glands of *Oncopeltus fasciatus*, a phytophagous insect employed as an experimental model [21], [28].

The present study investigated the relevance of *T. cruzi* cruzipain in the interaction process with *R. prolixus* midgut. Our results showed that the five cysteine peptidase inhibitors used (cystatin, antipain, E-64, leupeptin and iodocetamide) significantly decreased the adhesion of *T. cruzi* to *R. prolixus* posterior midgut. *T. cruzi* possesses two major cysteine peptidases, the cathepsin L cruzipain [15], [25], and a 30-kDa cathepsin B like-peptidase [22]. We showed that a specific cathepsin B inhibitor, CA-074-OMe, promoted no significant alteration in the adhesion rate, which is in accordance to its intracellular localization [22]. These findings suggest that a cathepsin L may be the molecule responsible for the reduced parasite binding to the insect midgut.

In plants and mammals, endogenous inhibitors of the cystatin superfamily are regulators of cysteine peptidase activity of enzymes from the papain superfamily with high affinity for cathepsin L. Chagasin, identified in *T. cruzi*, is a tight-binding high affinity reversible inhibitor of papain-like cysteine peptidases. In *T. cruzi*, chagasin interacts with cruzipain, regulating the activity of this enzyme [10], [30]. Herein, we showed that parasites treated with cystatin displayed reduced adhesion to the luminal surface midgut of the *R. prolixus* in a dose-dependent manner. Therefore, despite of the doubtful selectivity of some inhibitors, the results obtained using cystatin strongly suggest that papain-like peptidases are required for efficient interaction between the parasite and the insect midgut. Moreover, the blockade of cruzipain by antibodies also led to a significant reduction in the capacity of adhesion to the midgut of the insects in relation to untreated parasites. This effect may be caused by steric intervention, where the antibodies prevent the access of insect gut molecules to specific sites in cruzipain, which could act as a recognition molecule. It has been shown in several monoxenic trypanosomatids that the metallopeptidase gp63 participates in parasite attachment to the insect midgut through a proteolytic-independent mechanism, which involves the recognition and binding to a yet unidentified insect midgut receptor [23], [26]. Our results indicate that the pre-incubation of the *R. prolixus* midgut with cruzipain did not influence the interaction rate. This could be an indication that cruzipain does not act as an adhesion molecule, suggesting that it could act cleaving off either surface molecules from the parasite or the insect epithelial cells, thus exposing hidden relevant molecules, which may facilitate parasite access to binding sites, promoting adhesion and colonization. For instance, our group has previously shown that *P. serpens* cruzipain-like molecules are able to degrade *O. fasciatus* salivary gland proteins [31].

However, the lack of inhibition by the pre-treatment of the insect midgut with the purified enzyme (either active or inactive) cannot rule out the participation of cruzipain as a ligand/adhesion molecule. The attachment of microorganisms to a biological surface is a complex process involving specific interactions between adhesins and complex receptors on host tissues. It should be kept in mind that surface molecules do not exist in their isolated form in cellular systems. Experimental models describing structural and functional aspects of proteins have historically used purified molecules, mutants lacking genes coding for the enzymes, and specific protein-binding probes, including antibodies, peptides and inhibitors. These classic approaches have traditionally focused on isolated molecules for structural and/or functional testing [32]. However, these approaches do not take into account the molecular associations at the cell surface, for instance, protein-protein, lipid-protein, glycan-protein and all possible inter-associations. The study of isolated molecules is insufficient to fully elucidate the functional impact of the complex structures that can be formed and are upon influence of the microenvironment of the insect midgut.

It is well known that *T. cruzi* presents remarkable genetic diversity between isolates [33]. Distinct expression profile and activity either of the enzyme (cruzipain) or the inhibitor (chagasin) could contribute to the biologic heterogeneity found between different isolates of the *T. cruzi*. Tissue-culture trypomastigotes (TCT) from the G isolate are less infective to mammalian cells and presents reduced activity of cruzipain [9], [10]. In this sense, we sought to compare the ability of several *T. cruzi* isolates as well as chagasin transfectants to adhere to *R. prolixus*. The interaction rate of the chagasin transfectants to the insect midgut was considerably lower in relation to wild-type parasites. The addition of exogenous active cruzipain partially restored in a dose-dependent manner the adherence of chagasin overexpressing parasites to the insect midgut, which further supports that this reduction in the adhesion rate was linked to the reduced levels of cruzipain and not to other phenotypic alterations induced by the overexpression of chagasin. Also, *T. cruzi* G isolate adhered to the insect gut to a lesser extent in comparison to Dm28c, Y and CL Brener isolates, indicating a biological deficiency of the G isolate, possibly linked to the lower activity of cruzipain. In epimastigotes, chagasin and cruzipain co-localize in at least two compartments of the secretory pathway of epimastigotes: the Golgi complex and the reservosome. It was also shown that cruzipain-chagasin complexes are formed in living parasites. Therefore, it is conceivable that chagasin associates with cruzipain molecules in the Golgi complex before sorting to reservosomes, flagellar pocket and plasma membrane [10], [30], suggesting that the surface cruzipain is inactive/inaccessible in the chagasin transfectants. Also, it is still unknown if epimastigote surface cruzipain is proteolytically active or not [25]. Interestingly, it has been shown in other microorganisms that classical cytosolic enzymes are on the surface, present activity and act as an adhesin, even if these enzymes do not possess the classical N-terminal sequence that predicts surface location [34]. Accordingly, our research group has shown that *T. cruzi* calpain molecules, which are typical cytosolic proteins, are present on *T. cruzi* surface and are involved in the metacyclogensis, interaction with mammalian cells, parasite proliferation and adhesion to the insect vector [35]–[37]. Ultimately, it illustrates that the interaction process involves a pool of molecules both on the microorganism and the host.

The data from the *ex vivo* assays are very suggestive of the involvement of cruzipain in the interaction with the invertebrate host. This hypothesis was further supported by *in vivo* colonization experiments, which revealed parasites only in the rectum by direct microscopic counts. The traditional parasite quantification method, through direct microscopic observation, is exhaustive, subjected to errors, and with reduced sensitivity. This led us to develop, for the first time, a methodology using quantitative real-time PCR (qPCR) using SYBR-green targeting *T. cruzi* satellite-DNA to quantify *T. cruzi* infection in *R. prolixus*. qPCR assays performed with chagasin transfectant or wild-type parasites revealed that the ability of the former to colonize *in vivo* was drastically reduced, being detected both in the midgut and rectum at considerably lower levels than wild-type parasites, which were more abundant in the rectum. The rectal portion is considered a site of stress, which induces the parasite metacyclogenesis in the vector. It is worth mentioning that cruzipain is abundantly expressed on the surface of epimastigote forms, while in tissue culture-derived trypomastigotes, the surface labeling is either absent or faint [5]. This fact together with the hypothesis that cruzipain might be involved in the attachment to the insect midgut could help to explain why *T. cruzi* is released after metacyclogenesis. However, tissue-derived and insect-derived trypomastigotes may present distinct surface properties, and this should be further investigated.

It is well known that long periods of *in vitro* culture reduce the expression of parasite virulence factors. In this sense, we showed through flow cytometry analysis a considerable increase in the levels of *T. cruzi* surface cruzipain after colonization of *R. prolixus* rectum, in comparison to cells cultivated in BHI medium. Previous studies indicated that an attenuated strain of *Leishmania major* produced low amounts and low enzymatic activity of gp63. After serial passages of these parasites through either *Phlebotomus duboscqi* or through mice, the recovery of the proteolytical activity was seen in a similar level of that presented in a virulent strain of *L. major* [38]. Reduced levels of gp63 are frequent in *Leishmania* promastigotes that undergo long-termed maintenance *in vitro* [38]. In addition, our research group reported previously the enhancement in the expression of gp63-like molecules in *Herpetomonas samuelpessoai* after colonization of an insect host model, *Aedes aegypti* [39]. Attenuated *T. cruzi* strains display reduced content of active cruzipain compared to virulent strains [40], which suggests a strong correlation between the virulence/attenuation of long-term *T. cruzi* cultures and the activity of cruzipain.

Although cruzipain is defined as the major cysteine peptidase detected in *T. cruzi* epimastigotes, this peptidase is a member of a large multigene family composed of polymorphic genes, whose expression are stage regulated in the parasite [25]. In epimastigotes, the majority of cruzipain RNA encodes highly similar isoforms, while in trypomastigotes and amastigotes, the expression of more divergent cruzipain genes can be detected [41]. The majority of the biochemical studies on cruzipain were performed using the natural enzyme purified from epimastigotes. The major isoform isolated from epimastigotes has been referred as cruzipain 1 (n-cruzipain 1) [42]. Cruzipain 2 is preferentially expressed by trypomastigotes and amastigotes. Although the isoforms show distinct substrate preferences, which would implicate on unique functions [42], both n-cruzipain 1 and n-cruzipain 2 may participate cooperatively in relevant biological processes such as host cell signaling and invasion by *T. cruzi* [8], [9], as well as in the interaction with the insect host.

Altogether, these findings establish that cruzipain is one of the molecules involved in the interaction between *T. cruzi* and its invertebrate host. Indeed, our results demonstrated that this enzyme is involved in the successful adhesion to the epithelial cells of insect vector both *ex vivo* and *in vivo*, although the exact molecular mechanism should be further explored. Collectively, our work adds new insights, never assessed before, about the relevance of cruzipain in the infection of the insect vector, *R. prolixus*.
